# Restrictions of anthelmintic usage: perspectives and potential consequences

**DOI:** 10.1186/1756-3305-2-S2-S7

**Published:** 2009-09-25

**Authors:** Martin K Nielsen

**Affiliations:** 1 Department of Large Animal Sciences, Faculty of Life Sciences, University of Copenhagen, Denmark

## Abstract

Given the increasing levels of anthelmintic resistance in equine parasites, parasitologists now recommend traditional treatment approaches to be abandoned and replaced by more sustainable strategies. It is of crucial importance to facilitate veterinary involvement to ensure that treatment decisions are based on parasitic knowledge. Despite recommendations given for the past two decades, strategies based on the selective therapy principle have not yet been implemented on a larger scale in equine establishments. In contrast, treatment regimens appear to be derived from recommendations originally given in 1966. The province of Quebec in Canada, and an increasing number of European countries, have implemented prescription-only restrictions on anthelmintic drugs. Denmark introduced this legislation ten years ago, and some evidence has been generated describing potential consequences. It is without dispute that Danish veterinarians are now deeply involved with parasite management in equine establishments. However, little is known about the impact on levels of anthelmintic resistance and the risk of parasitic disease under these circumstances. In addition, the legislation makes huge demands on diagnosis and parasite surveillance. No data have been published evaluating fecal egg count techniques and larval culture methods as clinical diagnostic tools, and very little is known about potential correlations with actual worm burdens. This article provides a general review of anthelmintic strategies currently used in equine establishments and outlines the recommendations now given for parasite control. Preliminary experience with prescription-only restrictions in Denmark is presented and current research needs to further evaluate this approach are discussed.

## Introduction

In 1966, Drudge and Lyons [1] were first to describe a modern equine anthelmintic program based on suppressive treatments. The first benzimidazole type drugs had recently entered the market, and with these modern safe and broad spectrum drugs, a whole new approach could be taken. Drudge and Lyons used the best scientific evidence available at the time to suggest a treatment protocol involving treatment of all horses every other month year-round. This protocol has since then been denoted the interval dose regimen.

Drudge and Lyons identified *Strongylus vulgaris *as the primary target of the program given its high pathogenic potential, and they used knowledge about life-cycles and egg reappearance periods to determine treatment intervals. In 1966, anthelmintic resistance in equine parasites was already recognized on a single-case basis [2-4] but was not considered a potential generalized problem in horse establishments. It was therefore considered a solid approach to treat all horses with fixed year-round treatments to suppress egg shedding and thereby reduce parasite transmission to a minimum. Drudge and Lyons also suggested rotating between drug classes to ensure that all parasite groups were targeted in the treatment regimen.

With the advent of new drug classes such as pyrimidines (pyrantel) in the 1970s and avermectin/milbemycins (ivermectin and moxidectin) in the 1980s, more anthelmintic classes were now available. In the sheep industry, rotation between drugs was now recommended as a strategy to counteract development of anthelmintic resistance [5], and this principle was quickly adopted in the horse industry as well. The drugs available were now all broad spectrum, so the purpose of the rotation was no longer to ensure a targeting of all parasite groups, but entirely to prevent anthelmintic resistance. However, no experimental evidence suggests that this is feasible, and one study suggests the opposite [6].

Presently, cyathostomin populations have been reported widely resistant to benzimidazole drugs and increasingly resistant to pyrantel formulations [7,8]. In addition, *Parascaris equorum *isolates have been reported resistant to ivermectin [9-16] and moxidectin [9,13,15]. One study confirmed a purportedly resistant Canadian isolate by experimentally infecting foals and subsequently euthanizing them after blinded ivermectin treatments of half the foals [17]. Most recently several studies have reported that egg reappearance periods in cyathostomins after ivermectin treatment have been shortened from about eight weeks to about four weeks in the US [18], Brazil [19] and Germany [12]. With a critical test study, Lyons and coworkers recently illustrated that the shorter egg reappearance in cyathostomins appear to be due to immature stages in the intestinal lumen now surviving treatments, which indicates ivermectin resistance on that particular stage [20].

This clearly illustrates a need for a change in strategy. Treating all horses with frequent intervals year-round is considered the major reason for the current levels of anthelmintic resistance. With the ready availability of cheap and safe over-the-counter anthelmintic products, the veterinarian is now rarely involved with parasite control, and treatment decisions are now made without considering the biology of the parasites.

Parasitologists have warned about this increasingly for the past two decades, and several reports have advocated for more veterinary involvement [7,21,22]. Today, there is general agreement that anthelmintic strategies should maintain adequate levels of parasite refugia through a less intensive anthelmintic program [7,8,23-25]. The most consistently recommended strategy is selective therapy (targeted treatments), which is based on a principle of selecting individuals in the herd for treatment and leave the rest untreated. The basis for this is the well-described over-dispersion of parasites in hosts [26-28], where a minority of animals harbours the majority of parasites. With the trichostrongyle *Haemonchus contortus*, a highly pathogenic blood-sucking abomasal nematode of ruminants, this principle has been practised by evaluating mucosal pallor as an indication of anaemia levels caused by the parasites. This system known as FAMACHA^® ^[29] has proven highly applicable for small ruminants in areas where *Haemonchus contortus *predominates [30-33], but less useful in an area with lower prevalence of this parasite [34]. Other proposed methods for selecting "wormy" individuals are based on body-condition scoring or weighing animals on regular bases, and treating those with weight-loss or less satisfactory weight-gain [24].

With horses, recommendations of selective therapy are based on fecal analysis from all horses on the premises, and treatment of those exceeding a predetermined cut-off value. Pioneering scientists published preliminary studies already in 1991 suggesting that a control strategy based on the selective therapy principle was a valid approach [35,36]. Since then, other equine publications have followed up on the principle and provided some evidence illustrating the usefulness of this approach [37-39]. The rationale for this treatment principle is that individual horses have a strong tendency to consistently remain at the same level of egg shedding over time [36,39,40]. Thus, a minority of horses will consistently be shedding the majority of eggs in the population, while a majority remains at very low or even undetectable egg count levels. The choice of cut-off value for treatment typically lies in the range of 0-500 eggs per gram (EPG) with 200 EPG being the most often used [48,60]. However, no studies have been performed evaluating these choices from a health risk assessment or parasite control point of view, and such studies are needed to further develop and evaluate this approach.

## Anthelmintic strategies currently used

Several questionnaire surveys have illustrated parasite control strategies in the past decade and these are summarized in Table 1. Altogether these publications suggest a general trend of relatively frequent treatments without considering the size and composition of the worm burden. The majority of these surveys have no mention of usage of fecal egg counts, and only a couple of surveys report that less than 1% used these as a means of surveillance [46,47].

**Table 1:** Recent questionnaire surveys reporting the number of annual anthelmintic treatments in equine establishments

**Country**	**Annual treatments**	**References**
United Kingdom	6	[41-44]
Ireland	8-12	[21]
South Africa	5-7	[45]
Sweden	3.2	[46]
USA	4 or more	[47]

In summary, the large body of evidence indicates that the interval dose regimen is still the basic principle used in equine establishments in countries representing three different continents. Thus it is fair to assume that the above-mentioned recommendations of reducing the treatment intensity and basing treatments on parasite surveillance instead of treating prophylactically have not yet reached the equine industry, and the principle of selective therapy appears to be not used at all. Instead a treatment regimen, which was proposed in 1966 is considered the standard treatment method. This clearly illustrates a fundamental problem; scientific information generated by parasitologists does not appear to get disseminated to the horse industry. Reasons for this are probably several, but it has been identified as a general problem that veterinarians are no longer involved in the parasite control programs.

## Prescription-only restrictions

One way to ensure veterinary involvement is to make drugs available on prescription-only premises. In Europe, the trend goes towards such conditions in several countries, because of a recent European Union (EU) directive. This directive is very complex but the underlying principle is to prevent over usage of drugs in the livestock industry. Thus, veterinary drugs can only be applied by trained personnel, and only after a condition being diagnosed by a veterinarian. Technically, horses are regarded as production animals like cattle and swine, and the rules include anthelmintic drugs. As a result, over recent years Sweden, the Netherlands and Finland have implemented prescription-only restrictions on anthelmintic drugs, and more EU countries are likely to follow. In Denmark, such restrictions were implemented already 10 years ago, and this country has thus generated valuable experience with this legislation. In addition to this, the province Quebec in Canada has had similar legislations since the 1980s, which is exceptional in North America.

In Denmark, however, some information has been gathered. In 1995, a questionnaire survey was performed among Danish horse owners describing their approaches for parasite control [22]. At that time, anthelmintic drugs were still available over the counter, and the treatment approaches employed were similar to those described in other countries. On average, Danish horse owners treated foals, young horses and adult horses 4.3, 4.0 and 3.7 times annually, respectively. Horse owners used 2.4 different drug types per year with only limited fecal egg counts being performed.

In 1999, the prescription-only legislation came into effect in Denmark. The increasing levels of anthelmintic resistance in parasitic nematodes led Danish legislators to take this step. The legislation applies to both large and small animals, including horses, and the overall aim was to encourage veterinary involvement to reduce anthelmintic usage and development of drug resistance.

In 2004, a questionnaire survey was performed among Danish equine veterinary practitioners [48]. The aim was to describe how the prescription-only conditions had affected veterinarians in their approach to parasite control. A number of notable findings were made. Overall, equine practitioners appeared to be largely involved with parasite control. Ninetyseven % of the respondents were performing fecal egg counts on a routine basis and 41% also performed larval cultures for identifying large strongyle species. The primary parasite management was performed within the active grazing season with the majority of fecal sampling and anthelmintic treatments being performed in the spring and fall, while very little activity occurred in the winter months. A majority of practices used the selective therapy principle and cut-offs for treatment centered around 200 eggs per gram feces. Because of this an estimate of yearly number of treatments per horse was not possible to achieve due to the fact that it would depend on the actual fecal egg count in each horse. Altogether, however, the survey indicated that for adult horses most practitioners used a two-samples-per-year approach, while younger horses appeared to get treated on additional occasions.

## Danish experiences

The clearly different treatment scenario in Denmark gives rise to at least two questions; 1) Has the selection pressure for anthelmintic resistance been reduced in Denmark, and 2) can adequate parasite control be achieved under the drug restrictions? Although more studies are definitely needed to fully answer these questions, a few have been performed over recent years, and some indication of the answers can be found.

Earlier studies performed in Denmark had indicated pyrantel resistance [49], while signs of ivermectin resistance in *Parascaris equorum *was documented in one horse herd [11]. In 2008, we performed two surveys evaluating anthelmintic resistance in Danish horse farms. One study evaluated pyrantel embonate, and the other ivermectin. The studies will be published in separate reports but the main findings are summarized below.

### Pyrantel efficacy

This study has been presented at the World Association for the Advancement of Veterinary Parasitology (WAAVP) Meeting in 2009 [50]. In brief, 64 horse farms with a total of 1644 horses were investigated using the fecal egg count reduction test (FECRT). Horses shedding at least 200 strongyle eggs per gram feces pretreatment were included in the FECRT. Overall, mean farm efficacies ranged from 80% to 100%, with only one farm having an efficacy lower than 80%. Fifty-six farms had efficacies above 90%, and nine were in the range of 80-90%. Although none of the farms were experiencing treatment failure, this study suggests that pyrantel resistance is present in Denmark, despite the legislation. However, selection for resistant parasite strains may have occurred prior to 1999. Thus, in the study by Craven et al. [49], slight indications of emerging pyrantel resistance were found, and the drug restrictions may have been introduced too late to effectively delay further development of resistance. In addition, pyrantel embonate paste was recently found to be routinely used by two thirds of Danish equine practitioners [48].

Studies performed in countries surrounding Denmark have suggested emerging pyrantel resistance as well. In Sweden, pyrantel efficacies were found in the range of 95-100%, but the high variability led to wide 95% confidence limits on some farms, which raised suspicion of developing resistance [51]. Similar findings were recently made in Germany, where efficacy levels were 92-100%, but confidence intervals very wide [12]. However, these two studies used a McMaster technique with a detection limit of 50 eggs per gram (EPG), where low post treatment egg counts will remain undetected. Thus, such an egg count technique will largely overestimate drug efficacy for horses with moderate egg counts. The Danish 2008 study used a modified McMaster technique with a detection limit of 20 EPG, so efficacy levels cannot be compared across these studies.

In summary, it remains unknown whether Danish drug restrictions have affected pyrantel efficacy, and more evidence is needed to make any solid conclusions.

### Ivermectin efficacy

This study has been presented at the World Association for the Advancement of Veterinary Parasitology (WAAVP) Meeting in 2009 [52], and a full manuscript is in preparation. FECRTs were performed on 196 horses from farms practicing selective therapy. Efficacy against *P. equorum *was evaluated on 79 of these horses and was found to be 96% across all farms. Strongyle efficacy was 100% on all the farms. Egg reappearance periods (ERP) were investigated on nine of these farms with a total of 96 horses. Weekly FECRTs found ERPs to be at least six weeks on all the farms. In conclusion, this study illustrated no signs of ivermectin resistance in strongyle parasites, and an overall 96% efficacy against *P. equorum *as well. This is in contrast to a German study, where a strongyle ERP of five weeks was reported [12]. Ivermectin resistance in *P. equorum *has been documented in several European countries [9,12,14,16] including Denmark [11]. The present study suggests that ivermectin most likely still remains efficacious against *P. equorum *on a majority of farms, even in countries where resistance has previously been reported on a case-basis.

### Potential adverse effects of selective therapy

Very little evidence exists evaluating the long-term effects of anthelmintic treatment programs using selective therapy, and most statements made at this point will be speculative. However, one focus in Denmark is the prevalence of *S. vulgaris*, and it has been hypothesized that the big proportion of horses receiving little or no treatments due to their constantly low egg counts would allow this parasite to become more prevalent. This would be of concern, since *S. vulgaris *is known to be very pathogenic. Because of this, we included individual larval cultures in the pyrantel study mentioned above [50] to account for the prevalence of this parasite. As such, 1644 individual larval cultures were performed and 5% were found positive. On the farm level, 31 of the 64 farms had at least one horse positive for this parasite.

Historically, *S. vulgaris *was reported to be virtually 100% prevalent in individual horses [52-57], so in this context a prevalence of 5% cannot be claimed dramatic. However, it becomes relevant to investigate if Denmark has significantly higher levels of *S. vulgaris *than countries without prescription-only conditions. A Swedish abattoir survey performed in the mid 1990s reported 6.1% of 461 horses with lesions in the cranial mesenteric artery, and subsequent larval cultures from these horses yielded a *S. vulgaris *prevalence of 3.6% [58]. At the time of this study, anthelmintics were still available over-the-counter in Sweden, but yet the *S. vulgaris *prevalence was at the same level as in the Danish 2008 study. In addition, a questionnaire survey performed in 2003, revealed that Swedish horse owners treated without performing egg counts and with the annual number of treatments per horse averaging 3.2 [46]. This is similar to Danish treatment conditions prior to 1999 [22]. Limited information exists on Danish *S. vulgaris *prevalence in the years before 1999. One study performed in 1996 reported 11 of 56 Danish farms positive, but larval cultures were done by pooling feces from several horses in each culture [49]. Such composite cultures are most likely to underestimate the prevalence, since the few large strongyle larvae will be overwhelmed by the many cyathostomin larvae from negative horses in each culture. Thus, a direct comparison with the present study cannot be made. Hence, it remains unresolved whether Danish treatment restrictions have led to higher *S. vulgaris *prevalences, and presently no conclusions can be made either way.

## Discussion

Prescription-only restrictions represent one way of ensuring veterinary involvement in the anthelmintic strategies in livestock animals. The Danish experiences strongly suggest that veterinarians have taken a lead role in equine establishments, and as such the legislation can be determined to be successful. But several new challenges have arisen from this, and there is a strong need for scientific studies providing information needed to meet these challenges.

In a system, where treatments are performed on a prescription basis, high demands are made to the diagnostic tools available. Although the same basic coprological techniques have been used for the past 100 years, there is very little knowledge about their performance as equine diagnostics. A number of publications report comparisons of various egg count techniques and focus on detection limits, accuracy and repeatability [59-62], but no studies have been published reporting analytical sensitivities and specificities of these techniques. For diagnostic tests being used routinely in clinical practice, this is highly needed. In addition, fecal egg counts are often claimed to suffer from poor correlations with actual worm burdens and a couple of studies report raw data suggesting this [63,64], but so far no statistical evaluations of this relationship have been published. In addition, it becomes increasingly important to account for parasite burdens on a species level to better the clinical implication of any given egg count. Larval cultures are widely used in Denmark, but like egg count techniques this technique has never been validated as a diagnostic tool for equine usage. Thus, we don't have any information on the reliability of, for example a *S. vulgaris *negative culture. It is perceived that the diagnostic sensitivity is low, since even cultures with *S. vulgaris *larvae are largely dominated by cyathostomins. In addition, the larval culture method is time-consuming and laborious. Recent advances with molecular techniques detecting DNA from *S. vulgaris *have shown promise [65,66], but have not yet been made available for equine practitioners.

The large degree of veterinary involvement also makes huge demands on knowledge in the veterinary community. Horse owners often complain over conflicting messages when they ask different veterinarians for parasite advice, and there is a constant need for continuing education. Thus, there is a considerable gap in knowledge that needs to be filled. In Denmark, equine parasitology has been an integrated part of the course package for continuing education for veterinary practitioners over the past decade, and most certainly this has improved the general level of knowledge. Parasite control can be regarded a specialty area within veterinary medicine, and some Danish practices have specialized themselves in running fecal samples and providing advice on performing parasite control as their primary source of income. This trend could potentially become common in other countries as well.

Although anthelmintic strategies based on the selective therapy principle have been recommended for two decades, no long term evaluations have been performed. Thus, we lack knowledge on the following areas: 1) Rate of development of anthelmintic resistance, 2) Prevalence levels of specific parasite species of particular pathogenic potential, 3) Risk of parasitic disease, and 4) Other objective parameters such as body condition/weight gain. Studies evaluating these parameters are highly needed for equine parasite control to move forward. As mentioned above, the selective therapy approach is one way to employ a more sustainable treatment principle. However, other methods may be developed in the future. One such example is the concept of mosaic treatments, which has been developed for insecticides [67]. The principle is to apply treatments with different pharmacological products to different parts of the host population. Theoretically, this should ensure adequate parasite refugia to each drug type. As such, mosaic treatments represent an alternative to the traditional drug rotations or combination treatments, and experimental evidence evaluating this approach in horses should be highly welcomed.

## Conclusion

Prescription-only restrictions have led to increased veterinary involvement, and reduced anthelmintic treatment intensity in Denmark. How these treatment conditions affect levels of anthelmintic resistance and the overall health of horses on the longer term remains unknown. Emphasis is being put on the performance of diagnostic tools, and more data is needed to fully validate the techniques used presently.

## Competing interests

The author declares that they have no competing interests.
